# A Multifaceted Approach for Evaluating Hepatitis E Virus Infectivity In Vitro: Cell Culture and Innovative Molecular Methods for Integrity Assessment

**DOI:** 10.3390/vetsci10120676

**Published:** 2023-11-27

**Authors:** Tatjana Locus, Ellen Lambrecht, Sophie Lamoral, Sjarlotte Willems, Steven Van Gucht, Thomas Vanwolleghem, Michael Peeters

**Affiliations:** 1 Fisheries and Food, Technology and Food Unit, Flemish Research Institute for Agriculture (ILVO), Brusselsesteenweg 370, 9090 Melle, Belgium or tatjana.locus@student.uantwerpen.be (T.L.); ellen.lambrecht@ilvo.vlaanderen.be (E.L.);; 2 Sciensano, Infectious Diseases in Humans, Viral Diseases, Engelandstraat 642, 1180 Ukkel, Belgium; 3 Laboratory of Experimental Medicine and Pediatrics, Viral Hepatitis Research Group, University of Antwerp, Drie Eikenstraat 655, 2650 Edegem, Belgium

**Keywords:** Hepatitis E virus, cell culture, capsid integrity, genome integrity, PtCl_4_, RNase A, infectivity, long-range RT-qPCR

## Abstract

**Simple Summary:**

In Western countries, Hepatitis E virus (HEV) can infect humans when they consume contaminated food. In most cases, the infection resolves on its own with mild symptoms. However, for some individuals, especially those with weakened immune systems, it can lead to severe and long-lasting health problems. Currently, primary detection of the virus RNA in food is performed, but it does not allow for distinguishing between infectious and inactivated viruses. Another approach consists in detecting virus growth in vitro, but it is presently slow and requires specific skills. All these render the assessment of effectively inactivated HEV challenging. In our research, we explored (1) in vitro HEV cultivation methods and (2) alternative molecular methods that can distinguish between intact and damaged viruses. These newly developed methods will help to assess the virus infectivity along the food chain, thereby identifying risk factors and mitigation strategies (e.g., food processing techniques) enhancing food safety.

**Abstract:**

Hepatitis E virus is a prominent cause of viral hepatitis worldwide. In Western countries, most infections are asymptomatic. However, acute self-limiting hepatitis and chronic cases in immunocompromised individuals can occur. Studying HEV is challenging due to its difficulty to grow in cell culture. Consequently, the detection of the virus mainly relies on RT-qPCR, which cannot differentiate between infectious and non-infectious particles. To overcome this problem, methods assessing viral integrity offer a possible solution to differentiate between intact and damaged viruses. This study aims at optimizing existing HEV cell culture models and RT-qPCR-based assays for selectively detecting intact virions to establish a reliable model for assessing HEV infectivity. In conclusion, these newly developed methods hold promise for enhancing food safety by identifying approaches for inactivating HEV in food processing, thereby increasing food safety measures.

## 1. Introduction

Hepatitis E virus (HEV) is a major cause of viral hepatitis worldwide, with a notable prevalence of over 20,000 human acute clinical cases reported in Europe during the past decade [1,2]. While a considerable number of HEV infections remains asymptomatic, both acute self-limiting hepatitis and chronic cases in immunocompromised individuals, such as organ transplant recipients, are observed [3].

HEV is a quasi-enveloped single-stranded, positive-sense RNA virus with a length of around 7.2 kb [4,5]. The virus belongs to the *Hepeviridae* family, *Orthohepevirus A* species [6]. So far, eight HEV genotypes (gt) have been identified, five of which are reported to cause human disease [7]. Genotypes 1 and 2 infect only humans, causing epidemics via the fecal–oral route in developing regions, mainly due to water contamination. In contrast, genotypes 3, 4 and 7 exhibit a broader host range and are zoonotic, often associated with consumption of contaminated food (i.e., products containing pork, wild boar, deer or camel) [8,9].

In Europe, the majority of detected cases are locally acquired HEV gt3 infections, exceeding the number of imported cases linked to genotypes 1 and 2 [1,10,11]. The virus circulates asymptomatically in swine and wild boar with a high prevalence [12,13]. Its transmission is considered zoonotic since HEV RNA has been detected in pigs at the different stages of their production (i.e., liver, stool and blood samples of pigs bred in farms, in slaughterhouses (i.e., livers, surfaces and containers) and in food products ready for sale such as pork liver pâté and sausage) [14,15,16,17,18,19,20,21]. HEV RNA has also been detected in wastewater from pig farms and slaughterhouses [11]. Additionally, the infectivity of commercial food products has been demonstrated by challenge experiments in pigs and cell culture models [22,23,24], and direct epidemiological links have been found between human cases and consumption of HEV-contaminated food products [25,26,27,28]. Despite this, limited information is available regarding the virus’ survival during food processing [29,30,31,32,33]. Given these concerns, the need for robust and widely applicable techniques for detecting infectious HEV in environmental and food samples has been intensified.

Propagation in cell culture is the gold standard to examine viral infectivity, but HEV is notoriously difficult to grow, often requiring an adaptation of viral strain and host cell line, which has hindered the study of this virus [22,24,34,35]. Up to now, multiple cell culture models have been developed to isolate and propagate HEV [34,35]. While they gave promising results, the developed methods are complex, laborious and time-consuming, limiting their application in routine analyses and impairing the exploration of inactivation methods [36]. This highlights the necessity for a reliable and consistent cell culture model for HEV.

Due to the challenges associated with culturing the virus, the detection of HEV has predominantly relied on molecular techniques, particularly real-time reverse transcriptase quantitative polymerase chain reaction (RT-qPCR). While RT-qPCR offers rapid, sensitive, specific, reliable, quantitative and cost-effective detection of viral RNA in food and the environment, it fails to discriminate between infectious (i.e., intact) and non-infectious (i.e., damaged) particles in samples [36,37,38,39]. This is due to the fact that RT-qPCR detects and amplifies small parts of a viral genome that can originate from infectious viruses, but also from non-infectious viral particles with damaged capsids and/or genomes. As a consequence, there is a potential overestimation of the amounts of infectious virus present in a given sample, meaning that any consecutive conclusion must be taken cautiously [36,40,41]. This limitation underscores the necessity to develop improved methods that determine viral infectivity more realistically [42].

To overcome this problem, methods like capsid integrity techniques have been introduced. With these techniques, intact and non-intact viral capsids are differentiated by combining RT-qPCR with biochemical pre-treatments. Consequently, only viral RNA targets originating from intact viral particles—and not free nucleic acids or those inside damaged capsids—are amplified.

Platinum compounds have recently been used to evaluate virion integrity of viruses such as SARS-CoV-2, norovirus, hepatitis A virus and HEV [41,43,44,45,46,47,48,49,50,51,52]. However, the efficiency of the applied technique varies across viruses, virus concentration, virus sample and inactivation techniques. As a result, species-specific optimization is necessary.

An alternative method for assessing capsid integrity involves employing RNases as a pretreatment before nucleic acid extraction. RNases degrade “free” RNA, thereby enhancing the likelihood of detecting viral RNA originating only from intact viral particles. This method has been tested with varying degrees of success on several viruses such as enteroviruses, rotaviruses, human norovirus, hepatitis A virus and HEV [44,53,54,55,56,57,58,59,60].

However, a drawback of these capsid integrity assays is their inability to distinguish viruses with intact capsids but damaged genomes, for example due to UV disinfection [50,51]. This limitation arises from the reliance of capsid integrity assays on RT-qPCR for final RNA detection. Another approach is to assess viral genome integrity through a modification of the RT-qPCR method, aiming to detect longer genome fragments, a technique known as long-range RT-qPCR, thereby increasing the chances of identifying genome damage [36]. Successful amplification by long-range RT-qPCR suggests an intact RNA strand, indicating potential infectivity of the virus. Long-range RT-qPCR has been developed for adenovirus, murine norovirus and human norovirus [44,61,62,63,64,65] but has not yet been reported for HEV.

This study aims at addressing the above-mentioned challenges by (1) analyzing and adjusting currently available HEV cell culture models, so as to come to an easy and robust optimized model to asses HEV infectivity; and (2) developing virus integrity RT-qPCR-based assays that selectively detect HEV particles with an intact capsid and genome. By improving molecular detection and virus infectivity assays, this study contributes to the enhancement of food safety measures and provides a valuable tool for assessing infectious HEV along the food chain and associated risks.

## 2. Materials and Methods

### 2.1. Viruses

The used viruses originated from

(1) Supernatants collected every seven days from persistently HEV gt3 47832c straininfected A549 cells generously provided by Prof. Dr. Johne (The German Federal Institute for Risk Assessment, Germany) [29,66];
(2) A leftover viremic serum sample from a patient acutely infected with HEV gt3c (National Reference Center for viral hepatitis, Sciensano, Belgium).

All the samples were conserved at −80 °C until use.

### 2.2. HEV Cell Culture Infection Experiments

#### 2.2.1. Cell Lines and Media

Different cell lines were used, including human hepatocellular carcinoma cell lines HuH7 (CLS Cell Lines Service), HuH7-S10-3 (Dr. Farci, National Institutes of Health, USA), PLC/PRF/5 (ATCC), HepG2 (ATCC) and HepG2-C3A (ATCC). Additionally, human lung adenocarcinoma cell lines A549 (ATCC) and A549-D3 (Prof. Dr. Johne, The German Federal Institute for Risk Assessment, Germany), which are a derivative of A549 with heightened susceptibility to HEV gt3c [29,67], were tested. Finally, animal-derived cell lines N2A (mouse neuroblastoma, ATCC) and PK15 (porcine kidney, ATCC) were used.

Cultivation of A549, A549-D3 and PK15 cells was performed in minimal essential medium (MEM, Thermo fisher, Waltham, MA, USA, #31095-029), whereas the other cells were cultivated in Dulbecco’s modified Eagle’s medium (DMEM, Thermo fisher, #41965-039). These media were supplemented with fetal bovine serum (FBS, Sigma-Aldrich, Darmstadt, Germany, #F7524), i.e., 5% for A549 and A549-D3 and 10% for all the other cell lines, sodium pyruvate (1 mM, Sigma-Aldrich, #S8636), penicillin (100 U/mL, Thermo Fisher, #15140122), and streptomycin (100 µg/mL, Thermo Fisher, #15140122). The cells were incubated at 37 °C with 5% CO_2_.

Components in the cell culture medium such as FBS concentration and addition of dimethyl sulfoxide (DMSO) can influence infection [68,69,70]. Because of this, various infection media were tested as outlined in Table 1.

#### 2.2.2. HEV Inoculation

Cells were seeded (1–5 10^5^ cells/mL) in 96-well plates (Sigma Aldrich, #M0812) in 100 µL maintenance medium per well at 37 °C and 5% CO_2_. After three days, the maintenance medium was removed, cells were washed once with PBS and inoculated with 200 µL infection media containing 10^8^ equivalent gt3 47832c HEV copies/mL. After an incubation of one hour at 37 °C, cells were washed three times with PBS and supplemented with 200 µL infection medium (Table 1). Cells were kept at 37 °C and 5% CO_2_ for the entire duration of the experiment.

#### 2.2.3. Immunofluorescence Assay

After collection of supernatants, cells were fixed with 80% acetone (VWR, Radnor, PA, USA, #20063.365) and washed with washing buffer (i.e., 0.01% Tween 20 (Sigma Aldrich, # 8221840500) in PBS). After 1 h blocking with 5% bovine serum albumin solution (BSA, Life technologies, Merelbeke, Belgium, #15561020), anti-HEV ORF2 antibody targeting the capsid protein (Merck-Millipore, Darmstadt, Germany, #MAB8002) was added at 1/100 for 1 h. After three washing steps with 0.01% Tween 20 in PBS, Alexa Fluor 488 Goat Anti-Mouse IgG (Life technologies, #A11029) was added at 1/400 along with DAPI (4′,6-diamidino-2-phenylindole, 300 nM, Thermo Fisher, # D1306) for 1 h. Finally, cells were washed three times with the washing buffer and three times with PBS. Cells were immediately viewed under an Olympus IX73 inverted fluorescence microscope.

A positive fluorescent signal was defined as (1) cells with a bright cytoplasm or (2) cells with more than five bright dots in the cytoplasm. A fluorescent signal outside the cells was not considered as a positive signal. The number of fluorescent foci was quantified with ImageJ software (version 1.54f) [71].

### 2.3. Capsid Integrity Assays

PtCl_4_ (Sigma Aldrich, #379840) was dissolved in DMSO (Sigma-Aldrich) at 50 mM, aliquoted and stored at −20 °C until use.

To assess the ideal concentration, PtCl_4_ was added to HEV RNA (90 µL) and to the HEV serum sample (90 µL) to reach final concentrations of 0.5, 1, 2.5, 5 and 10 mM. The samples were incubated for 30 min on ice while shaking at 300 RPM.

Similarly, RNase A (VWR, #E866) was added to HEV RNA (90 µL) and to the HEV serum sample (90 µL) to reach final concentrations of 10, 25, 50, 100, 250 and 500 µg/mL, and samples were incubated for one hour at 37 °C. The enzymatic reaction was halted by adding 140 U of RNase OUT (Thermo Fisher, #10154652) for 30 min at room temperature.

### 2.4. RNA Extraction, Purification and Detection

Following RNA sample treatment with PtCl_4_ and RNase A, viral RNA purification was performed according to the RNA Cleanup protocol from the RNeasy Mini kit (Qi-agen, #74106), according to the manufacturer’s instructions.

Viral RNA extraction was performed by using the QIAamp Viral RNA Mini Kit (Qiagen, #52906), following the manufacturer’s instructions.

HEV RNA was detected using a one-step RT-PCR protocol [72]. For each reaction, 5 or 15 µL of sample RNA was mixed with 15 µL of MasterMix, consisting of 1 µM of TaqMan™ Fast Virus 1-Step Master Mix (Thermo Fisher, # 5555534) and the following primers and probe: forward primer HEV-AB-F (5′-CGGTGGTTTCTGGGGTGA-3′, 0.5 µM), reverse primer HEV-AB-R (5′-GCRAAGGGRTTGGTTGG-3′, 0.5 µM), and probe HEV-pr-MGB (5′-FAM-ATTCTCAGCCCTTCGC-MGB-3′, 0.25 µM). Reverse transcription was performed at 50 °C for 5 min, followed by denaturation for 3 min at 95 °C and 45 cycles of 15 s at 95 °C and 30 s at 60 °C.

### 2.5. Genome Integrity Assay: Two-Step Long-Range RT-qPCR

For the development of the long-range RT-qPCR, several parameters and conditions were tested.

The reverse transcription (RT) step involved using either an oligo(dT)-VN (20T-VN IDT) primer or an HEV-specific primer HEV-AB-R (5′-GCRAAGGGRTTGGTTGG-3′) [72] to reverse transcribe RNA to cDNA, commencing near the 3′ poly(A) tail and extending towards the 5′ cap structure.

Three different reverse transcriptases (MultiScribe RT #4311235, SuperScript III RT #18080093, and SuperScript IV RT # 18090050 from Thermo Fisher) were assessed. Thermo Fisher’s RT protocols were followed, exploring various reaction parameters, including template HEV RNA quantities of 1, 5, or 7.5 µL, the presence or absence of 5% DMSO in the Master Mix, and incubation temperatures of 55 °C, 60 °C, and 65 °C.

qPCR primers targeting the 5′ end of the HEV genome were designed. To achieve this, complete HEV gt3 genomes available on the National Centre for Biotechnology Infor-mation (NCBI) website were selected and aligned (Clustal Omega, RRID:SCR_001591, on https://galaxy.pasteur.fr/ accessed 28 January 2022) to generate a consensus sequence. Then, qPCR primers and probes were designed with Primer Blast (https://www.ncbi.nlm.nih.gov/tools/primer-blast/ accessed 28 January 2022).

For TaqMan primer sets, tested reaction conditions (25 µL) included 1x TaqMan Environmental Master Mix (Thermo Fisher, #4396838), FW/RV primers (at concentrations of 50, 300, 500, 600, and 900 nM), a 250 nM probe, and 5 µL of HEV cDNA. The amplification protocol consisted of an initial activation step at 95 °C for 10 min, followed by 40 cycles of denaturation at 95 °C for 15 s, annealing at temperatures of 55 °C, 58 °C, 60 °C, 62 °C or 64°C for 1 min, and a final elongation step at 72 °C for 5 min.

Similarly, for SYBR Green primer sets, reaction conditions (25 µL) encompassed 1x SSO Advanced SYBR Green inhibitor-tolerant mix (Bio-Rad, Hercules, CA, USA, #1725016), FW/RV primers (250 nM), and 5 µL of HEV cDNA. The amplification protocol included an initial activation step at 98 °C for 2 min, followed by 40 cycles of denaturation at 98 °C for 15 s, annealing at temperatures of 55 °C, 58 °C, 60 °C, 62 °C or 64 °C for 1 min, and a final elongation step at 72 °C for 5 min. Within each run, standard curves were constructed using 10-fold dilution series (10^7^ to 10 genome copies/reaction) of the HEV WHO reference reagent (Paul Ehrlich Institute, Langen, Germany) and gBlock (IDT, 423 bp artificial HEV gt 3 DNA sequence consisting of part of ORF1) for quantification purposes.

### 2.6. Statistical Analysis

Data were processed and analyzed with Rstudio (R version 4.1.2 (November 2021)—R Foundation for Statistical Computing). Data were first tested for normality and equal variances by the Shapiro–Wilk test and Levene’s test, respectively. Normally distributed data were analyzed by parametric tests, i.e., *t*-test. The non-parametric equivalents (i.e., Wilcoxon rank-sum test and Kruskal–Wallis test) were applied in case of non-normal data. As a post-hoc test, Dunn’s (1964) Kruskal–Wallis multiple comparison test was performed to study differences between groups, and *p*-values were adjusted according to the Bonferroni method. A value of *p* < 0.05 was considered to be significant.

## 3. Results

### 3.1. Evaluation of HEV Cell Culture Models

#### 3.1.1. A549-D3 and HuH7-S10-3 Cells Are Promising for a Cell Culture-Based HEV Infection Model

A549, A549-D3, HuH7-S10-3, PLC/PRF/5, HepG2, HepG2-C3A, N2A, HuH7 and PK15 cell lines were inoculated with cell culture-derived HEV gt3c and maintained in MMEM-10-D at 37 °C and 5% CO_2_. Seven days post inoculation, cells were fixed and stained with an anti-HEV capsid antibody. At the same time, supernatants were collected and HEV RNA was detected by conventional RT-qPCR. As a baseline for the replicating RNA, supernatants were also collected at day zero.

**Figure 1:**
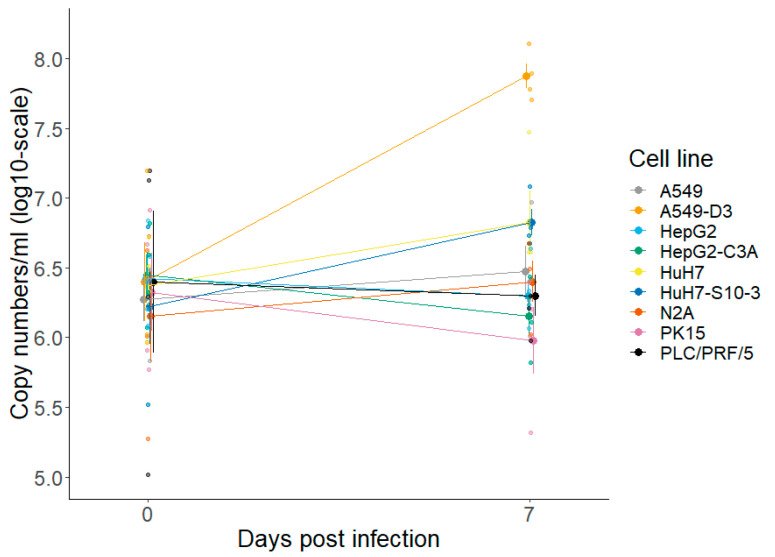
The HEV permissivity of all tested cell lines (i.e., nine in total) was assessed by conventional RT-qPCR on released viral particles in the supernatant seven days post-inoculation. Genome copies per mL are displayed on a log10-scale. Represented are means with SE (n = 4).

A549-D3 cells showed a significant increase of HEV RNA in the supernatant seven days after inoculation with HEV (Figure 1). Among all tested cell lines, they demonstrated the highest viral load. A numeric increase was also observed in HuH7 and HuH7-S10-3 cells, which did not reach statistical significance. However, viral concentrations in supernatant obtained from these cell lines never reached those from A549-D3 cells. The other cell lines seemed to be refractory to HEV infection.

**Figure 2:**
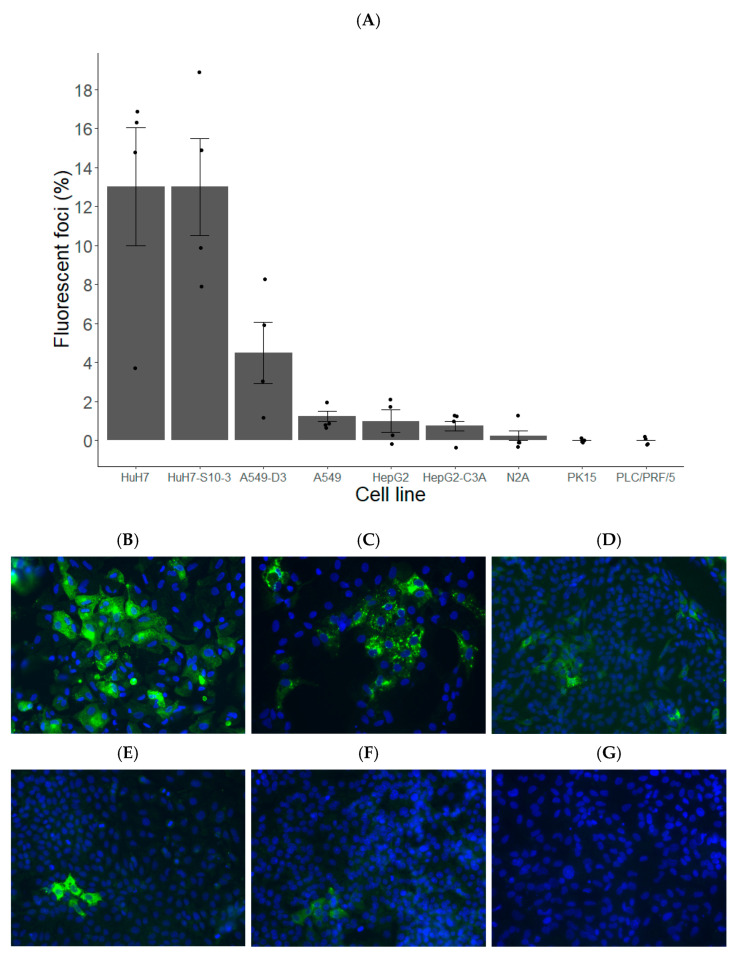
HuH7, its subclone HuH7-S10-3 and A549-D3 are promising cell lines for the analysis of HEV infectivity. (**A**) The average percentage of fluorescent foci was calculated (n = 4) per cell line as well as the SE. (**B**–**G**) The HEV permissivity of HuH7 (**B**), HuH7-S10-3 (**C**), A549-D3 (**D**), A549 (**E**) and HepG2-C3A (**F**) cells was assessed by HEV ORF2 immunostaining (in green) and nucleus (DAPI, in blue), with non-infected HuH7 cells (**G**) as a control on day 7 after inoculation.

HuH7 and HuH7-S10-3 showed the highest percentage of infected cells, followed by A549-D3, A549, HepG2, HepG2-C3A and N2A cells (Figure 2A–E). The other cell lines did not show any HEV infection.

Based on the results of the HEV capsid intra-cellular immunostaining and conventional RT-qPCR performed on supernatants of inoculated cells, it was decided to continue with A549-D3 and HuH7-S10-3 cells.

#### 3.1.2. FBS and DMSO Positively Contribute to HEV Propagation in A549-D3 and HuH7-S10-3 Cells

A549-D3 and HuH7-S10-3 cells were seeded in maintenance medium for three days and then inoculated with cell culture-derived HEV gt3c. After inoculation, infection medium was added for seven days. Infection mediums specified in Table 1 were tested. 

**Figure 3:**
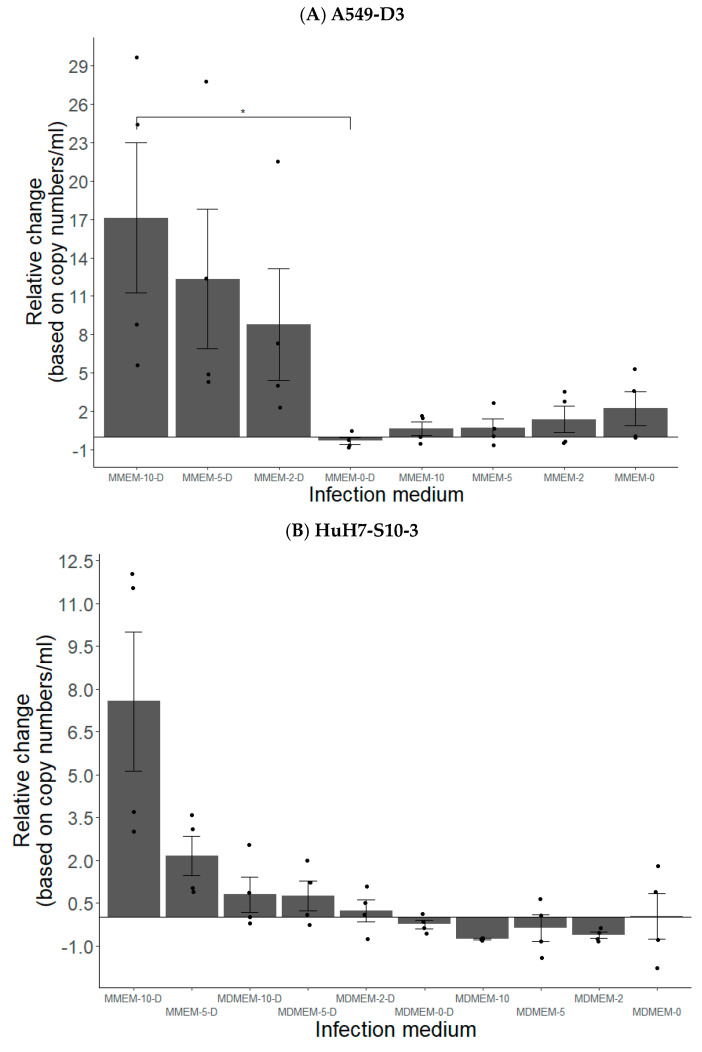
Conventional RT-qPCR performed on supernatants of A549-D3 (**A**) and HuH7-S10-3 (**B**) cells. Graph illustrates the proportional viral RNA change in cell culture supernatants 7 days after inoculation, in respect to residual viral RNA post 1 h inoculation washing based on copy numbers present per mL supernatants. Presented are mean barplots with standard error (n = 4). * *p* < 0.05.

Optimal viral production in supernatant of both cell lines was achieved by cultivating cells in MEM supplemented with 5% and 10% FBS along with 2% DMSO, denoted as MMEM-5-D and MMEM-10-D, respectively (Figure 3A,B). In addition, MMEM-based media resulted in higher infection rates compared to MDMEM-based media (Figure 3B). Strikingly, media with FBS but without DMSO and vice-versa led to lower HEV production compared to medium without FBS and DMSO, as shown by conventional RT-qPCR performed on cell supernatants (Figure 3A,B).

**Figure 4:**
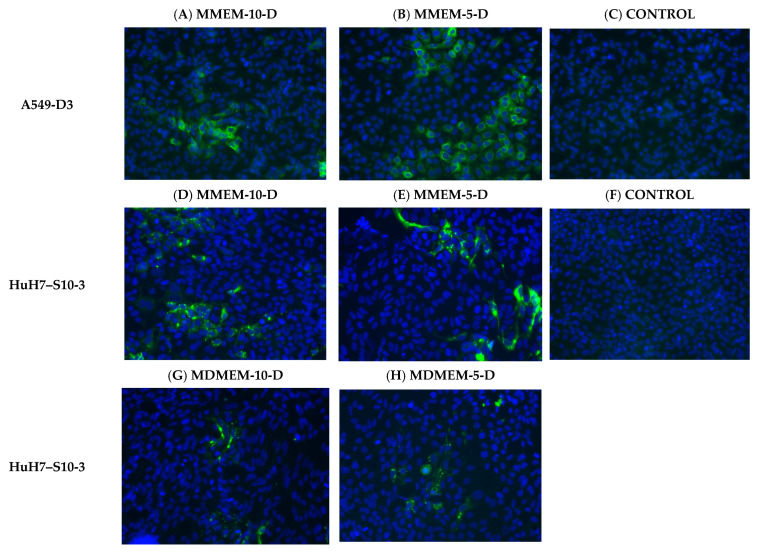

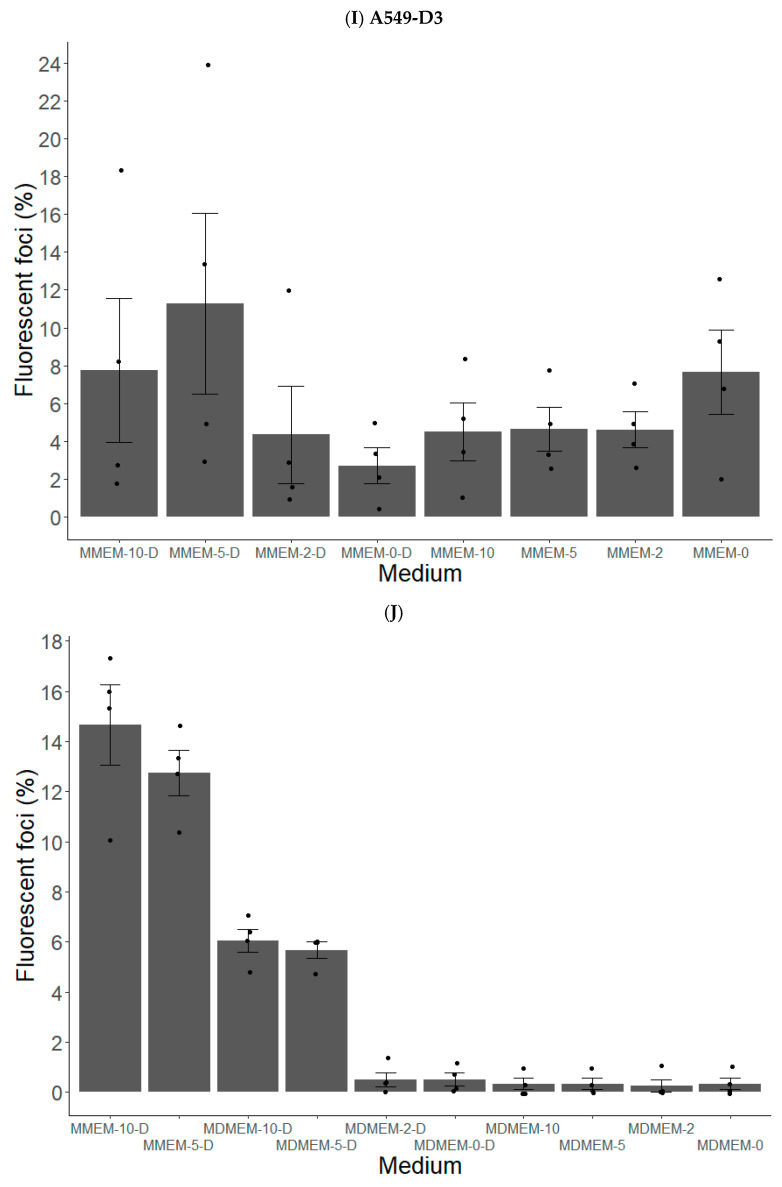
Cultivation of A549-D3 and HuH7-S10-3 cells in medium supplemented with combined FBS and DMSO favor viral propagation. (**A**–**H**) The HEV permissivity of inoculated cells was assessed by immunostaining of HEV ORF2 (in green) and nucleus (DAPI, in blue). A549-D3 cells grown in (**A**) MMEM-10-D and (**B**) MMEM-5-D, HuH7-S10-3 cells grown in (**D**) MMEM-10-D, (**E**) MMEM-5-D, (**G**) MDMEM-10-D, (**H**) MDMEM-5-D, with non-infected A549-D3 (**C**) and HuH7 (**F**) cells as controls. The average percentage of fluorescent foci was calculated (n = 4) for A549-D3 (**I**) and HuH7-S10-3 (**J**) cells per medium composition, as well as the SE.

Immunostaining outcomes were largely comparable to the viral production results (Figure 4A–J). The highest amount of positive cells was observed for cells that were cultured in MMEM-5-D and MMEM-10-D (Figure 4A,B,D,E,I,J), highlighting the synergic effect of serum and DMSO on HEV infection. Also, MMEM resulted in higher infection rates compared to MDEM (Figure 4D,E,G,H,J). Intriguingly, cultivation in MMEM-10-D did not yield the highest count of infected A549-D3 cells (Figure 4A,B), despite resulting in the highest viral production (Figure 3A).

Based on the results of the conventional RT-qPCR performed on supernatants of inoculated cells and of the HEV ORF2 intra-cellular immunostaining, it was decided to cultivate A549-D3 and HuH7-S10-3 cells in MMEM-10-D medium in the following experiments.

#### 3.1.3. The Peak of HEV Concentration in Supernatant of A549-D3 and HuH7-S10-3 Cells Is Reached after Five and Six Days, Respectively

A549-D3 and HuH7-S10-3 cells were seeded in maintenance medium for three days before being inoculated with the cell-culture-derived HEV gt3c. Infection lasted four, five, six or seven days in MMEM-10-D medium.

**Figure 5:**
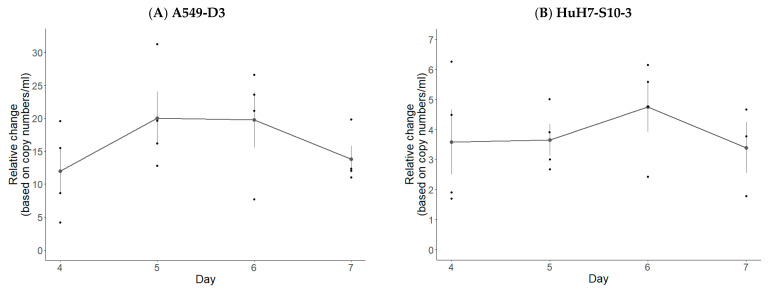
The peak of HEV RNA concentration in supernatant is reached at day five in A549-D3 cells (**A**) and at day six in HuH7-S10-3 cells (**B**) after HEV inoculation. Graph illustrates the proportional viral RNA change in cell culture supernatants at the end of the experiment (i.e., 4, 5, 6 or 7 days), with respect to residual viral RNA post 1 h inoculation washing based on copy numbers present per mL supernatants. Presented are means with standard error (n = 4).

The peak of viral RNA detection in supernatants occurred after five days for A549-D3 cells and six days for HuH7-S10-3 cells (Figure 5). A substantial mean increase of 20X with respect to residual viral RNA post 1 h inoculation washing was observed in A549-D3 cells, whereas HuH7-S10-3 cells exhibited a mean maximal increase of 4.5X. Both cell lines exhibited a decline in the detected viral RNA levels beyond day six.

Immunostaining outcomes mirrored this trend closely, revealing a similar pattern. The highest count of ORF2 positive cells was observed at day five for A549-D3 cells and day six for HuH7-S10-3 cells.

### 3.2. PtCl_4_ and RNase A Combined with Conventional RT-qPCR Can Be Used as HEV Capsid Integrity Assays

**Figure 6:**
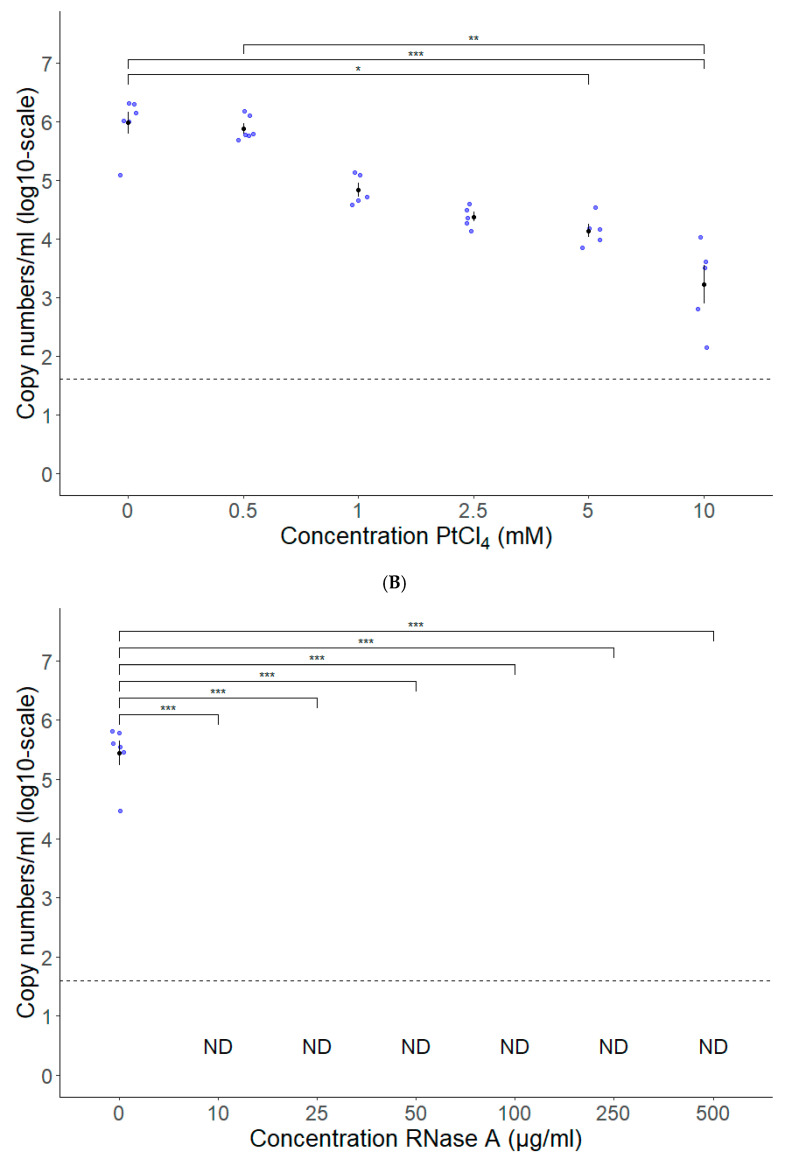
Capsid integrity assays. (**A**) PtCl_4_ treatment at 10 mM significantly reduced free HEV RNA detection. (**B**) RNase A treatment at 10 µg/mL is sufficient to reduce completely free HEV RNA detection. Means are displayed with standard error in black; individual datapoints are displayed in blue; dotted line is detection limit of the RT-qPCR. ND = Not Detected (n = 6). * *p* < 0.05, ** *p* < 0.01, and *** *p* < 0.001.

The optimal concentration of PtCl_4_ was determined by treating free HEV RNA obtained after nucleic acid extraction of an infected serum sample with PtCl_4_ at concentrations of 0.5, 1, 2.5, 5 and 10 mM prior to conventional RT-qPCR. The addition of PtCl_4_ significantly reduced the amplification from 5 mM, but no complete loss was observed at any tested concentration with an average reduction of 98.7% and 99.7% at 5 mM and 10 mM, respectively (Figure 6A). Importantly, no significant differences in RNA detection was observed when HEV-positive serum samples were treated at any tested concentrations, indicating that PtCl_4_ does not alter the detection of HEV RNA supposed to be protected by a capsid (Supplemental Figure S1A). It was decided that 10 mM PtCl_4_ was the ideal concentration.

As a second method for the proxy-detection of infectious virions, RNase A followed by conventional RT-qPCR was used. Extracted HEV RNA was treated with RNase A at concentrations of 10, 25, 50, 100, 250 and 500 µg/mL in order to determine the optimal concentration. Figure 6B shows a complete loss of detection of RNA as soon as 10 µg/mL RNase A is added (Figure 6B). In HEV-positive serum samples treated at any tested concentration, no significant differences in RNA detection was observed, indicating that RNase A treatment does not alter the detection of HEV RNA protected by a capsid. (Supplemental Figure S1B). It was decided that 10 µg/mL RNase A was the ideal concentration.

### 3.3. Long-Range RT-qPCR Can Be Used as a Proxy for Detecting Intact HEV Genomes

To develop the long-range RT-qPCR, various parameters and conditions were systematically tested and evaluated.

For the reverse transcription of HEV template RNA, the specific primer HEV-AB-R (5′-GCRAAGGGRTTGGTTGG-3′) proved to be more effective than an oligo(dT)-VN (20T-VN IDT) primer [72]. Reverse transcription using MultiScribe RT and SuperScript III RT did not yield satisfactory results, prompting further optimization efforts involving SuperScript IV RT and a 7.5 µL template volume. Optimal results were attained by incorporating 5% DMSO during the incubation step at 60 °C (Supplementary Table S1).

For the qPCR part, eight primer pairs located near the 5′ end of the HEV genome were tested. They are presented in Table 2.

Following HEV DNA PCR amplification, which was confirmed by observing the correct size and clearly visible signal through gel electrophoresis, a subset of four primer pairs (i.e., 1, 2, 6, and 7) was selected from the initial eight, based on their performance across five distinct annealing temperatures. These selected primer pairs underwent evaluation via qPCR encompassing three different annealing temperatures (specifically, 60 °C, 62 °C, and 64 °C) (Supplementary Table S2). Based on the assessment of qPCR efficiency and slope, two primer pairs (i.e., 1 and 6) were deemed suitable and retained for further investigation.

For primer pair 1, a comprehensive evaluation encompassed multiple primer concentrations (ranging from 50 to 900 nM) in conjunction with annealing temperatures of 60 °C and 62 °C (as detailed in Supplementary Table S3). Ultimately, a primer concentration configuration of 600 nM for the forward primer and 900 nM for the reverse primer, coupled with an annealing temperature of 60 °C, proved the best condition (Table 3).

In the case of primer pair 6, an optimal configuration was determined by selecting an annealing temperature of 60 °C along with a primer concentration of 250 nM (Table 3).

For further experiments, it was decided to use the long-range RT-qPCR with primer pair 1.

## 4. Discussion

Hepatitis E virus is a foodborne pathogen, and its prevalence in Europe has been on the rise. However, the lack of a validated and user-friendly cell culture model has hindered HEV infectivity and inactivation studies. Simultaneously, there is a need for alternative, faster molecular methods to estimate virus infectivity. In this study, our objective was to optimize an easily accessible cell culture model while simultaneously developing capsid (PtCl_4_ and RNase A) and genome integrity (long-range RT-qPCR) assays to evaluate HEV integrity as a proxy for infectivity.

A comparison of the developed HEV detection methods is provided in Table 4.

In order to apply the cell culture model for future large-scale evaluations of HEV contamination after food processing, we aimed to select the most suited cell line for HEV cultivation, examine various infection conditions and minimize the total duration of the cell culture model.

A higher proportion of HEV ORF2-positive foci were observed in HuH7 and its subclone HuH7-S10-3, with less ORF2-positive foci in A549, A549-D3, HepG2 and HepG2-C3A cells. N2A, PK15 and PLC/PRF/5 cells exhibited minimal to no positive foci. When looking at viral RNA detection in supernatants, A549-D3 had a significant higher level than the other cell lines. We chose to focus on HuH7-S10-3 and A549-D3 cells because they had the highest percentage of ORF2-positive cells and the highest viral RNA production, based on immunofluorescent staining and RT-qPCR, respectively.

To expedite the cell culture assay, we adopted shorter seeding durations of three days and higher cell densities, ensuring high confluence at the time of infection [74,75]. This choice was based on the practicality of using this model for rapid food safety assessments.

In addition to the cell line used, other factors can also have an effect on infection. Previous studies have identified serum and DMSO as factors influencing in vitro HEV replication [68,69,70]. Our study demonstrated that optimal viral infection in both HuH7-S10-3 and A549-D3 cell lines was achieved when cultured in MEM supplemented with 10% FBS, along with 2% DMSO. DMSO is known to impact cell growth, survival, differentiation and viral infection [68,70]. Its addition can induce differentiation or polarization in hepatocyte cell lines, potentially enhancing infection by certain viruses such as hepatitis B, C, and E [68,76,77,78,79,80]. Previous studies reporting increased HEV production with DMSO supplementation mainly utilized human hepatoma cell lines [68,70,78]. However, we observed significantly higher viral RNA levels in supernatants of A549-D3 (i.e., human lung adenocarcinoma) cells compared to HuH7-S10-3 cells. One hypothesis is that DMSO might induce cellular stress, rendering cells more susceptible to HEV infection. This hypothesis gains support from the observation that addition of FBS (i.e., 5–10%) in combination with DMSO was necessary to achieve high levels of viral RNA in our study. FBS, commonly added to cell cultures to enhance cell proliferation [81], could potentially indirectly prevent the adverse effects of DMSO, resulting in cell rescue but also in higher infection rates. However, the influence of FBS on infection is multifaceted, either inhibiting or promoting infection [29,68,75,82,83,84,85]. Our observations demonstrated reduced viral production when FBS was added to the medium in the absence of DMSO, suggesting that FBS alone might impede HEV infection in A549-D3 and HuH7-10-3 cells.

We observed a significant increase in viral RNA in supernatants within just five days for A549-D3 cells and six days for HuH7-S10-3 cells by cultivating cells in MEM supplemented with 10% FBS and 2% DMSO during the infection period. It is noteworthy to mention that many established cell culture models for HEV typically employ more extended periods, ranging from seven days to even several months [29,68,75,86,87]. Moreover, these models often exhibit a slow initial phase of viral replication before substantial increases. In contrast, our model, particularly in the case of A549-D3 cells, demonstrated a relatively rapid onset of viral replication. This shortened duration offers new perspectives for exploring the presence of infectious HEV in food products by using cell cultures.

The final HEV cell culture model with A549-D3 and HuH7-S10-3 cells achieved high viral replication and infection rates in a significantly shorter evaluation period. However, it is important to acknowledge that even with these improvements, the process remains time-consuming and labor-intensive (Table 4). To mitigate these challenges, we simultaneously developed RT-qPCR based virus integrity assessment methods. Currently, RT-qPCR stands as the primary method for HEV RNA detection, but it lacks the ability to distinguish between infectious and inactivated viruses. The development of techniques capable of distinguishing between particles with intact capsids and/or genomes will enhance the reliability and utility of future RT-qPCR results. These methods provide an indirect way to estimate infectivity, within a shorter timeframe, potentially aiding in the timely evaluation of infectious HEV presence.

To estimate HEV capsid integrity, we evaluated two methods: RNase A and PtCl_4_ pretreatment. Both methods were able to significantly reduce (i.e., PtCl_4_) or completely inhibit (i.e., RNase A) the signal of free RNA, indicating that these methods could be used in the future to determine the capsid integrity of HEV.

In the literature, the efficacy of RNases to degrade RNA from inactivated viruses varies, with some studies reporting successful degradation [44,58,60], while others found less favorable results [56,57,88]. These discrepancies could be due to variations in RNase concentrations, incubation times, sample types, and virus types [46,56,59,60,62,88]. This highlights the need to optimize RNase pretreatment conditions for specific viruses and sample types. On the other hand, our experiments with PtCl_4_ revealed that none of the tested concentrations completely inhibited the amplification of free RNA. This outcome was somewhat unexpected, as previous studies had demonstrated PtCl_4_’s ability to completely inhibit the signal of free RNA and inactivated viruses, often at lower concentrations [43,44,46,89]. However, other studies, reported a reduction in signal without complete removal, consistent with our findings [41]. In contrast, Chen and colleagues observed no reduction in viral detection after PtCl_4_ treatment of heat-inactivated human noroviruses, possibly due to the low concentration that was applied (500 µM) [49]. Our study also found that this concentration was insufficient to reduce free RNA, emphasizing the importance of method optimization for obtaining reliable results. Nevertheless, our results suggest that PtCl_4_ treatment may possibly lead to an overestimation of particles (albeit small) with intact capsids, since none of the tested concentrations were able to completely remove the RT-qPCR signal from free RNA, indicating that this might also be the case with inactivated viruses (e.g., through food processing such as heating).

Lastly, we explored the possibility of PtCl_4_ and RNase A damaging intact viral capsids, as observed in other studies [44,60]. Our experiments demonstrated no reduction in viral RNA detection when membrane-associated, quasi-enveloped HEV from a patient’s serum was treated with PtCl_4_ or RNase A, compared to untreated samples. This indicates that even at the highest concentrations tested, RNase A and PtCl_4_ do not damage intact HEV capsids.

These developed methods for assessing capsid intactness provide a more accurate measure of viral integrity than traditional molecular methods alone, which will help differentiate between infectious and inactivated HEV particles.

In addition to capsid integrity, genome integrity plays a crucial role in determining viral infectivity. Certain inactivation methods, such as UV irradiation and chlorination, specifically target the viral genome without necessarily causing damage to the viral capsid. It has been demonstrated in previous studies that, in such cases, conventional RT-qPCR assays may not accurately estimate the quantity of viral particles with intact genomes [61,62,64]. In such scenarios, the utilization of long-range RT-qPCR is more appropriate, as these methods take into consideration the integrity of the viral genome. Notably, long-range RT-qPCR has proven to be considerably more accurate than conventional RT-qPCR in estimating remaining infectious virus following UV treatment, as demonstrated in the case of murine norovirus [61]. We developed for the first time a long-range RT-qPCR method for HEV detection. However, one limitation of the long-range RT-qPCR assay lies in its reliance on high-quality, undamaged template RNA for the reverse transcription step [66]. In our study, optimizing the RT step proved challenging and highly dependent on the quality of template RNA. Potential damages arising during viral RNA extraction, freeze-thaw cycles, pipetting and other processes may compromise RNA integrity, resulting in reduced quantities of intact cDNA available for qPCR amplification. Still, this innovative method offers the capability to identify viral particles with intact genomes, providing a valuable addition to the field of HEV research.

In summary, while the cell culture model provides a direct assessment of HEV infectivity, it remains time-consuming, albeit shorter than many existing models. Conversely, molecular methods for assessing HEV integrity offer speed but only serve as a proxy for infectivity. Capsid integrity assays excel in cases of damaged or destroyed capsids, while long-range RT-qPCR efficiently detects intact genomes. Therefore, a combination of the three approaches offers the most comprehensive assessment of HEV integrity and potential infectivity.

In the present paper, we exclusively assessed our methods against a cell culture- grown HEV strain and a human infected serum from the Belgian National Reference Center. Results observed with these strains might differ from those observed when food matrices are artificially or naturally contaminated. The efficiency of HEV detection in meat products could diverge due to the presence of fat, salt, protein and other components. Further research is ongoing to evaluate the power of our methods in assessing HEV infectivity in food matrices. Consequently, at present, these methods may not be readily applicable in current food safety protocols; however, they exhibit promise for future applications.

Moreover, exploring the power of the developed methods when testing various environmental conditions, such as wastewater and surfaces (e.g., cutting boards in slaughterhouses, hands of food handlers, etc.), represents a worthwhile direction. This exploration has the potential to enhance food safety at the farm level, aligning with the One Health principle.

Furthermore, the virus integrity-based detection techniques could serve as valuable alternatives for other foodborne viruses that are challenging to cultivate in cell culture, including HAV and norovirus. It is crucial to note, though, that achieving optimal performance would necessitate additional refinement.

Still, our study represents a significant step forward in optimizing HEV contamination assessment through the cell culture model and innovating virus integrity evaluation methods. These advancements hold the potential to expedite the detection of infectious HEV along the food chain and enhance the reliability of HEV research.

## Figures and Tables

**Table 1:** Infection media compositions.

Name	Composition
MMEM-0	Maintenance * MEM with 0% FBS
MMEM-2	MMEM-0 + 2% FBS
MMEM-5	MMEM-0 + 5% FBS
MMEM-10	MMEM-0 + 10% FBS
MMEM-0-D	MMEM-0 + 2% DMSO
MMEM-2-D	MMEM-2 + 2% DMSO
MMEM-5-D	MMEM-5 + 2% DMSO
MMEM-10-D	MMEM-10 + 2% DMSO
MDMEM-0	Maintenance * DMEM with 0% FBS
MDMEM-2	MDMEM-0 + 2% FBS
MDMEM-5	MDMEM-0 + 5% FBS
MDMEM-10	MDMEM-0 + 10% FBS
MDMEM-0-D	MDMEM-0 + 2% DMSO
MDMEM-2-D	MDMEM-2 + 2% DMSO
MDMEM-5-D	MDMEM-5 + 2% DMSO
MDMEM-10-D	MDMEM-10 + 2% DMSO

* Maintenance medium is the medium in which cells are grown, i.e., MEM or DMEM supplemented with 1 mM sodium pyruvate, 100 U/mL penicillin, 100 µg/mL streptomycin and FBS. The percentage of FBS supplemented and the addition of 2% DMSO to the media are mentioned by a number (i.e., 0, 2, 5, 10) and the letter D, respectively.

**Table 2:** Seven primer pairs were designed for this study. The last primer pair (pair 8) was designed based on conventional PCR primers from Fogeda and colleagues [73].

Pair	Target	Forward (FW) Reverse (RV) Probe (Pb)	Sequence 5′-3′	Start (Position)	Stop (Position)	Length (Nucleotides)
1	Pre-methyltransferase (MeT)	FW	CTACTGCCATTGAGCAGGC	68	86	126
		RV	GCTGCCGGGGTTGCATC	193	178	
		Pb	CTGTGGTGGTTCGGCCGTT	121	140	
2	Pre-methyltransferase (MeT)	FW	CTACTGCCATHGAGCARGC	68	86	126
		RV	GCTGCCGGGGTTGCATC	193	178	
		Pb	CTGTGGTGGTTCGGCCGTT	121	140	
3	Pre-methyltransferase (MeT)	FW	CTTGGCGAATGCTGTGGTG	111	129	83
		RV	GCTGCCGGGGTTGCATC	193	178	
4	Pre-methyltransferase (MeT)	FW	ACTACTGCCATTGAGCAGGC	67	86	80
		RV	GATAAAAACGGCCGAACCACCAC	146	124	
5	Pre-methyltransferase (MeT)	FW	GTGGTCGATGCCATGGAGG	19	37	128
		RV	GATAAAAACGGCCGAACCACCAC	146	124	
6	Y-domain & papain-like cysteine protease (PCP)	FW	CGYCAGCTYCAGTTTTATGC	1306	1325	173
		RV	CAGGTRCACTCCTGCCC	1478	1462	
7	Y-domain & papain-like cysteine protease (PCP)	FW	CGYCAGCTYCAGTTTTAYGC	1306	1325	173
		RV	CAGGTRCACTCCTGCCC	1478	1462	
8	methyltransferase (MeT) [73]	FW	CTCCTGGCRTYACWACTGC	56	74	172
		RV	GGRTGRTTCCAIARVACYTC	227	208	

**Table 3:** The optimal qPCR primers for the two-step long-range RT-qPCR, based on slope and efficiency of serial dilution of HEV DNA gblock (10^7^–10 genome copies/reaction).

Primer Pair	Concentration Forward Primer	Concentration Reverse Primer	MasterMix	Efficiency	Slope	Y-Intercept
1	600 nM	900 nM	TaqMan environmental	1.971	−3.394	33.75
6	250 nM	250 nM	SSO advanced SYBR Green inhibitor tolerant	1.971	−3.393	32.24

**Table 4:** Comparison of the developed HEV detection methods.

Parameter	PtCl_4_	RNase A	Long-Range RT-qPCR	Cell Culture
Infectivity assessment	Indirect (Capsid integrity)	Indirect (Capsid integrity)	Indirect (genome integrity)	Direct
Time	+++	+++	++	+
User friendly	+++	+++	+	+
Cost price	+++	++	+	+
Sensitivity	+++	+++	++	++

## Data Availability

The data presented in this study are available based on a motivated request sent to the corresponding author and after an MTA.

## Supplementary Materials

The following supporting information can be downloaded at: https://www.mdpi.com/article/10.3390/vetsci10120676/s1, Figure S1: PtCl4 (A) and RNase A (B) do not enter viral particles with intact capsids; Table S1: Set-up long-range RT-qPCR—RT conditions; Table S2: Set-up long-range RT-qPCR—Selected qPCR primers; Table S3: Set-up long-range RT-qPCR—qPCR primer concentrations.
