# Supplementary material for: A Multifaceted Approach for Evaluating Hepatitis E Virus Infectivity In Vitro: Cell Culture and Innovative Molecular Methods for Integrity Assessment
Source: Vet Sci. 2023 Nov 27;10(12):676. doi: 10.3390/vetsci10120676 (PMC10748075; doi:10.3390/vetsci10120676)
Supplement: Supplementary file 1 [file vetsci-10-00676-s001.zip › Supplementary Materials.pdf]

A

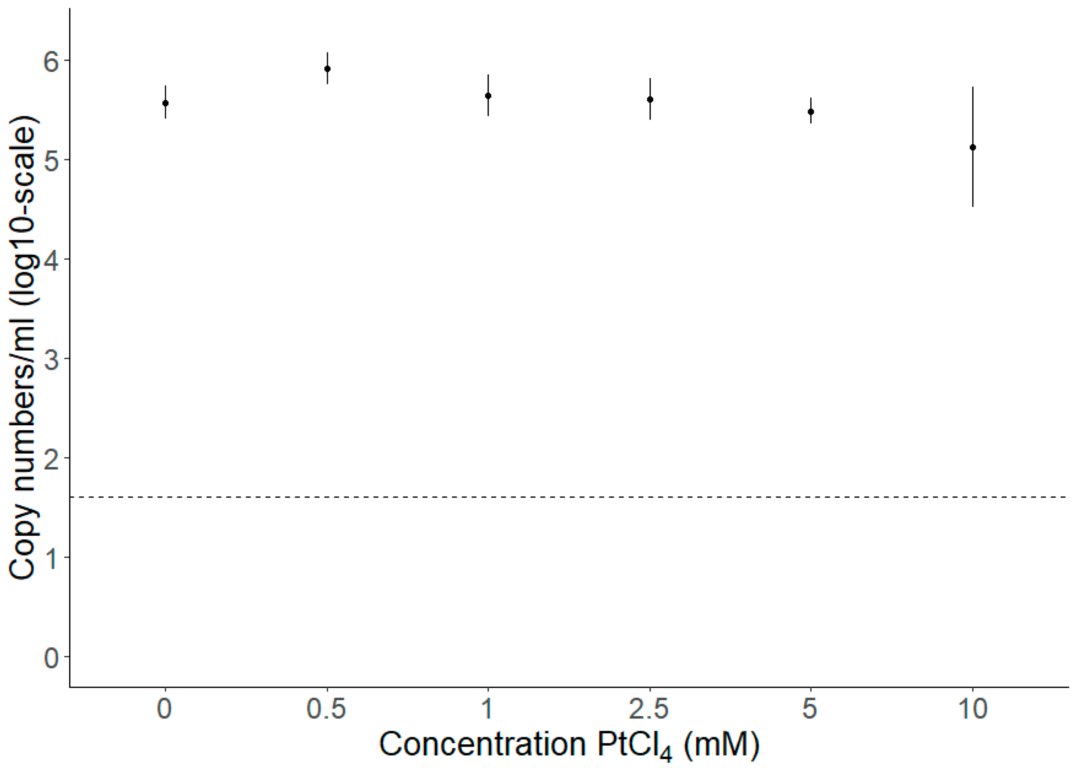

B

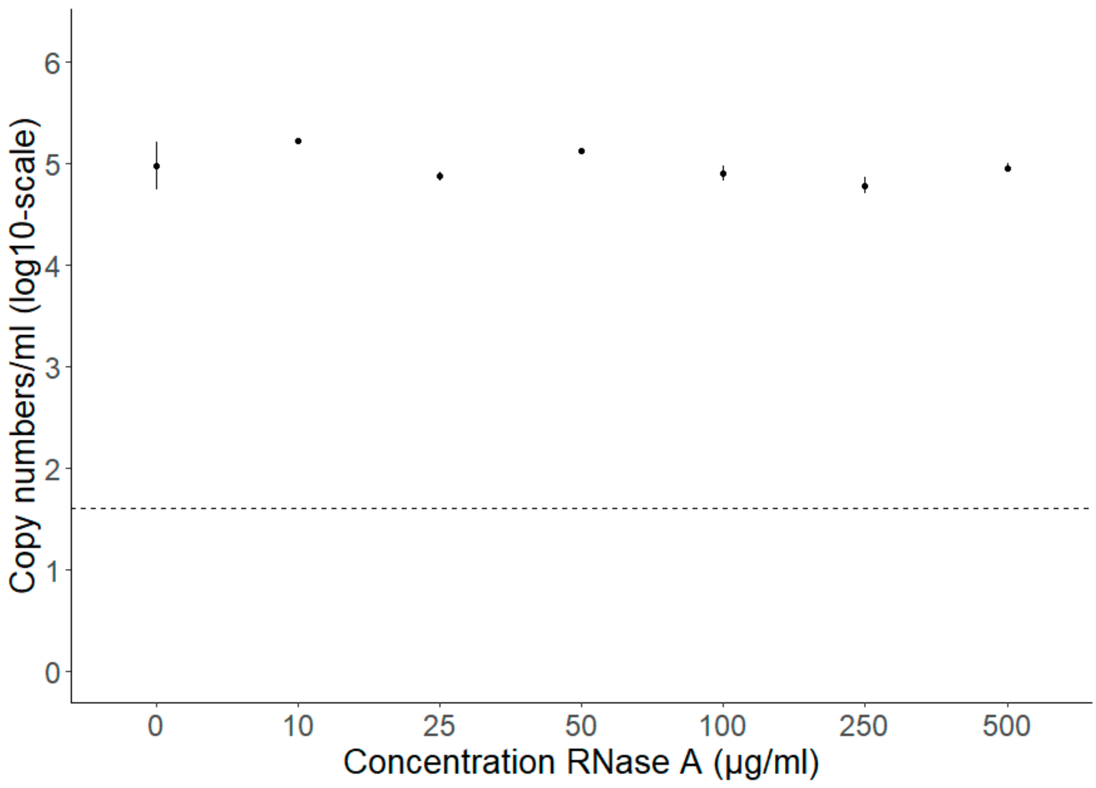

Figure S1: $PtCl_4$ (A) and RNase A (B) do not enter viral particles with intact capsids. No difference is observed in RNA detection when HEV positive serum samples are treated with different concentrations. Displayed as means and SE (n = 4 for $PtCl_4$ and 2 for RNase A). The dotted line represents the detection limit of the RT-qPCR.
